# Landscape of gene mutation in Chinese thyroid cancer patients: Construction and validation of lymph node metastasis prediction model based on clinical features and gene mutation marker

**DOI:** 10.1002/cam4.5945

**Published:** 2023-04-20

**Authors:** Meng Wei, Rui Wang, Wanxue Zhang, Jing Zhang, Qiang Fang, Zheng Fang, Bin Liu, Yongxiang Li

**Affiliations:** ^1^ Department of General Surgery The First Affiliated Hospital of Anhui Medical University Hefei China

**Keywords:** lymph node metastasis, nomogram, RET genetic alteration, thyroid cancer

## Abstract

**Objectives:**

Reporting the clinicopathological information of thyroid cancer (TC) patients from a central medical center of east China, and constructing the nomogram predicting lymph node metastasis (LNM).

**Methods:**

We collected the patients who underwent thyroid cancer surgery in our institute from July 1, 2019 to July 31, 2021, a total of 253 subjects were enrolled. We used HiPure FFPE DNA Kit to extract DNA and RNApure FFPE Kit to extract RNA from the paraffin sections of tumor tissue, the extracted DNA samples and RNA samples were used for NGS sequencing. The clinical and pathological information of TCGA‐THCA cohort was obtained as the validation cohort. Multivariate logistic regression analysis was performed to identify the independent prognostic factor, and the nomogram was subsequently constructed by “rms” R package.

**Results:**

Secondary cases contained more mutation of BRAF (90.48% vs. 62.07%) and TERT (33.0% vs. 3.0%), as compared with primary cases. Primary patients with positive lymph node were younger (40.9 ± 10.8 vs. 45.3 ± 11.8, *p* = 0.0031) and contained advanced TI‐RADS levels (4c: 22.8% vs. 8.3%, 5: 6.5% vs. 0/0%, *p* = 1.878e‐03), as well as more RET genetic alteration (16.3% vs. 2.7%, *p* = 2.566e‐03). We chose age, tumor diameter, RET fusion, and gender to construct the LNM predicting nomogram. Calibration plot, DCA curve, and the clinical impact plot verified the preferable prognostic value of the nomogram, with an AUC value of 0.724 (0.656–0.792). We successfully validated the prognostic value of the nomogram in TCGA‐THCA cohort. RET fusion might impact the process of protein digestion and absorption, cytokine‐cytokine receptor interaction, ECM‐receptor interaction, focal adhesion.

**Conclusion:**

We provide a novel nomogram to predict the LNM for TC patients, including the features of patient's age, gender, tumor diameter, and RET alteration. Further studies from multiple medical centers are essential to validate the nomogram.

## 1 INTRODUCTION

The incidence of thyroid cancer (TC), ranking ninth place for global incidence, has been rapidly ascending over the last few decades in most areas of the world.^1^, ^2^ Specifically, in China, from 2008 to 2012, about 47,550 new TC cases were diagnosed. The incidence rate of TC, reaching 7.56/100,000, has ranked the seventh in overall malignant tumors.^3^ From 2005 to 2015, the incidence of TC showed upward trend, increasing from 3.21/100,000 in 2005 to 9.61/100,000 in 2015.^4^ Differentiated thyroid cancer (DTC) is the most common subtype of TC,^5^ which includes papillary thyroid cancer (PTC) and follicular thyroid cancer (FTC), accounts for more than 90% of thyroid malignancies in the world.^6^ Surgery followed by radioactive iodine therapy or chemoradiation are both effective treatments and targeted treatment of the cancer for example BRAF inhibitors MEK inhibitors has also been a prospective method to cure of TC.^5^ Although majority of patients have favorable prognoses, with the 5‐year‐survival rate of about 98.2% in PTC patients (PTCP) around the world and 84.3% in TC patients in China,^7^, ^8^, ^9^, ^10^ lymph node metastasis (LNM) is still a risk factor which contributes to the poor prognosis of TC patients.^11^ Therefore, we aim to construct new models to predict LNM to optimize treatment of patients.

The detection rate of lymph node metastasis (LNM) is frequent with 13.4%–60% in PTCP.^8^ The study of Nie et al.^12^ also found that the rate of lateral LNM and central LNM were 74.9% and 70.5%, respectively, in PTC patients in China. Until now, univariate and multivariate analyses have both revealed LNM played a significant role in predicting disease recurrence.^11^ Presence of LNM can cause five times more likely to relapse than the absence of LNM.^11^, ^13^ The study of Lee et al.^14^ found that the deaths can be up to 16 patients, who died of distant metastasis or locoregional failure, among 18 patients with recurrence, nearly 89%.^14^ Therefore, we could consider LNM as a poor prognostic factor. Nowadays, for more accurate prognosis, many scientists have focused on some molecular marks. BRAF^V600E^ mutation and RET mutation are greatly common, with frequency ranging from 33.2% to 88% in PTC and 40% to 50% in patients with sporadic medullary TC, respectively.^8^, ^13^ BRAF^V600E^ mutation can lead to aberrant activation of the mitogen‐activated protein kinase, which will increase cell proliferation and differentiation, eventually causing the thyroid oncogenesis.^8^, ^15^, ^16^, ^17^ Moreover, BRAF^V600E^ mutation is correlated with the poor survival rate of PTCP and some poor clinicopathologic features, such as multifocality, tumor size, vascular invasion,^15^, ^17^ revealing it can be an important candidate biomarker for the prognosis of TC patients. RET mutation can cause tumor cell survival and proliferation, induce c‐cell transformation, finally exerting their oncogenic effects on the thyroid.^13^ Until now, many types of RET/PTC rearrangements have also been studied. RET/PTC1 is associated with excellent prognosis while RET/PTC3 is related with poor prognosis.^18^ Considering LNM and some molecular mutations are both associated with the prognosis of patients, so it is of interest to study the relation between them.

In this study, we gathered the clinicopathological information of TC patients from a central medical center of east China and identified the predict factors that can reflect the presence of LNM and finally constructed a nomogram that can predict LNM for TC patients.

## 2 METHODS AND MATERIALS

### 2.1 Collection of patients

We collected the patients who underwent thyroid cancer surgery in our institute from July 1, 2019 to July 31, 2021, a total of 253 subjects were enrolled. All the patients were diagnosed with unilateral or bilateral thyroid nodules of category 3 or higher by ultrasound, CT or MRI. The samples were further double checked with TC by intraoperative frozen section examination and postoperative pathological examination. We recorded the clinicopathological parameters of age, gender, tumor diameter, ultrasound TI‐RADS stage, with or without ultrasound abnormal node, with or without ultrasound abnormal calcification, tumor pathological type, tumor TNM stage, and with or without lymph node metastasis. The TNM staging was based on the staging definition criteria in the eighth edition of the American Joint Committee on Cancer (AJCC).

### 2.2 Inclusion and exclusion criteria

All the selected patients should meet the following criteria: (1) did not receive radionuclide iodine‐131 therapy or radiofrequency ablation before surgery; (2) between 18 and 75 years old; (3) without chronic diseases of hypertension, heart disease, diabetes, etc.; (4) cooperate with the follow‐up. Exclusion criteria: (1) without certain pathological diagnosis of TC; (2) with severe dysfunction of liver, kidney, heart, and lung; (3) not agree to receive NGS testing; (4) others who are considered inappropriate by the researchers. This study was approved by the Ethics Committee of the First Affiliated Hospital of Anhui Medical University, and written informed consent was obtained from the participants and the data involved were anonymous without any identifiable private information.

### 2.3 Sequencing of tumor tissue

We used HiPure FFPE DNA Kit to extract DNA and RNApure FFPE Kit to extract RNA from the paraffin sections of tumor tissue, the extracted DNA samples and RNA samples were used for library construction, and then the Qsep100 automatic nucleic acid and protein analysis system was used to assess the quality of the constructed library. For NGS sequencing, the steps are as follows: (1) thawing the sequencing reagent; (2) cleaning the instrument before sequencing; (3) cleaning the Flowcell; (4) establishing a Sample Sheet; (5) dilution and mixing of the library; (6) adding the library; (7) run sequencing; (8) clean the instrument after sequencing. SNV and Indel mutations were filtered with the follow criteria: (1) mutation frequency > 0.01; (2) population frequency (refer to the East Asian population database of 1000G and ExAC) less than 0.01; (3) retain the sites of exons or alternatively spliced regions, and retain the sites of the target UTR region (such as the TERT promoter region); (4) remove the sites of synonymous mutation; (5) remove background mutations and low‐frequency mutations in repetitive regions.

### 2.4 Bioinformatic analysis

The clinical and pathological information of TCGA‐THCA cohort was obtained from UCSC Xena, as well as the gene expression profile and gene mutation matrix (https://xenabrowser.net/datapages/?cohort=GDC%20TCGA%20Thyroid%20Cancer%20(THCA)&removeHub=https%3A%2F%2Fxena.treehouse.gi.ucsc.edu%3A443). The heatmap for the clinical information and waterfall plot for the distribution of gene mutation was completed with “ComplexHeatmap” R package.^19^ Multivariate logistic regression analysis was performed to identify the independent prognostic factor, and the nomogram was subsequently constructed by “rms” R package. Calibration curve, decision curve and clinical impact curve analysis were all performed to assess the clinical predict function and accuracy of the nomogram via the “rms” and “rmda” packages. Differentially expressed genes (DEGs) was calculated by “limma” R package, and “clusterProfiler” package^20^ was employed to annotate the enriched biological process, cell components and molecular function of GO terms, as well as the KEGG pathways.

### 2.5 Statistics

Student's *t* test was used to compare the distribution between two groups, and Fisher's exact test was performed to distinguish the difference of categorical data. All statistical analyses were performed by R (Version: 4.1.2). A two‐tailed *p* value <0.05 was recognized statistically significant.

## 3 RESULTS

### 3.1 Basic information and differences between primary and secondary cases of AHMU‐TC cohort

**FIGURE 1:**
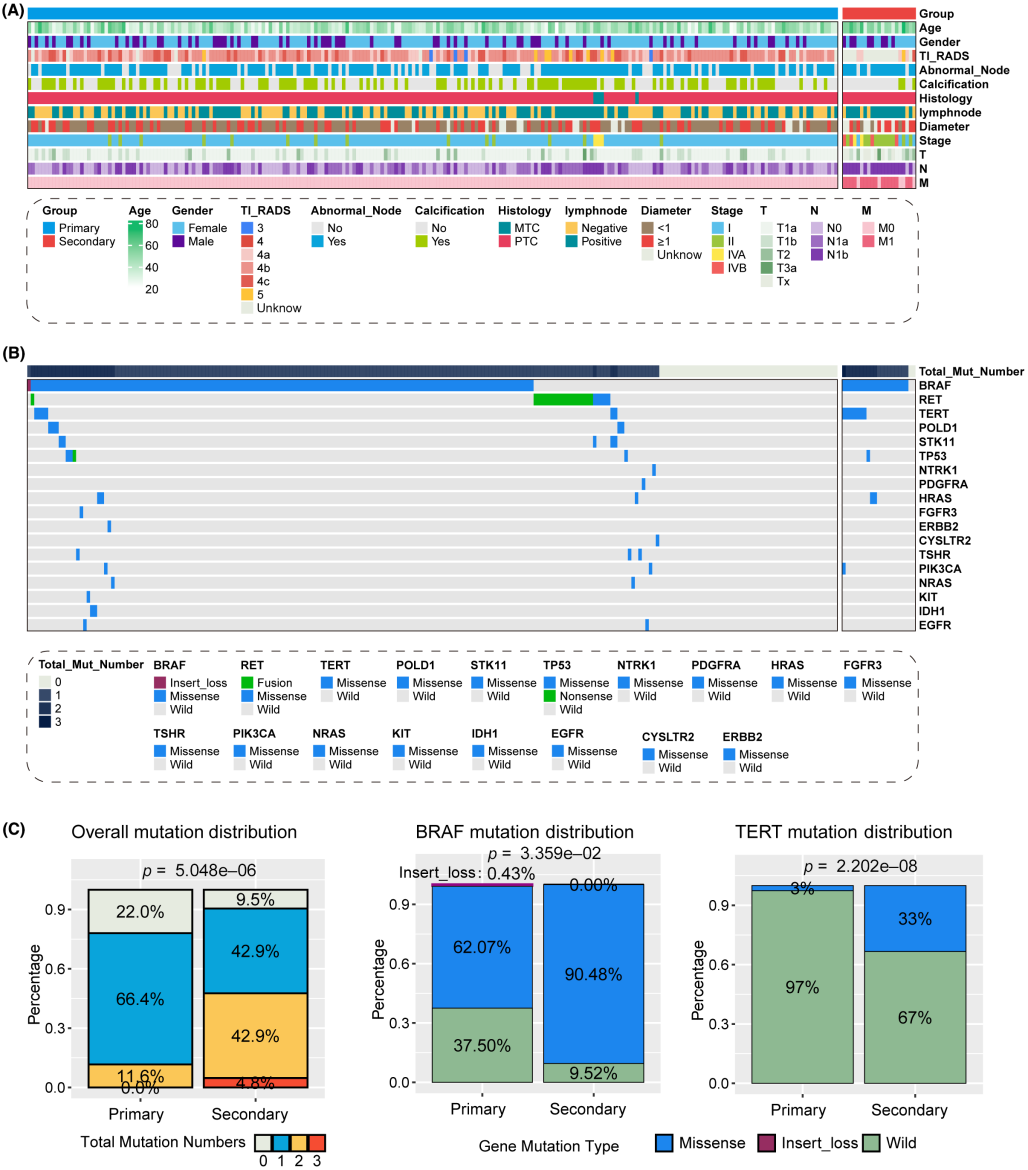
Analysis of the distribution of mutations in the AHMU‐TC cohort, mainly analyzing the differences in gene mutations and clinical indicators between primary and recurrent thyroid cancer patients. (A) Distribution of clinical and pathological information between primary and secondary thyroid cancer patients. (B) Differential distribution of gene mutations in patients with primary and secondary thyroid cancer. (C) Differential distribution of total mutation number, BRAF mutation and TERT mutation among patients with primary and secondary thyroid cancer.

With the pre‐set criteria, we collected a total of 253 TC patients from our institute, consist of 232 primary cases and 21 recurrent cases (also termed as secondary cases). The basic clinical pathological information of all the patients listed in Table 1 and Figure 1A. We first compared the different clinicopathological information between primary and secondary cases. We observed that the average age for secondary cases was higher than primary cases (51.5 ± 12.2 vs. 43 ± 11.4), and the tumor longest diameter in secondary group also showed a higher value (1.38 ± 1.2 vs. 0.958 ± 0.746). In addition, secondary group contained less ultrasound detected calcification (14.3% vs. 47.8%), but more lymph node metastasis (76.2% vs. 53.0%). We demonstrated the genetic alteration for all the 253 TC patients with the waterfall plot (Figure 1B), and several differences of genetic alteration was also displayed. Significantly, more secondary cases contained at least one mutant gene as compared with primary cases (90.5% vs. 88.0%, *p* = 5.048e‐06), and only one patient contained three types of mutant gene, BRAF, TERT, and PIK3CA, belonged to secondary group (Figure 1C). In addition, secondary cases contained more mutation of BRAF (90.48% vs. 62.07%) and TERT (33.0% vs. 3.0%, Figure 1C).

**TABLE 1:** Basic clinical features of enrolled patients.

	Primary (*N* = 232)	Secondary (*N* = 21)	Overall (*N* = 253)
Gender			
Female	163 (70.3%)	13 (61.9%)	176 (69.6%)
Male	69 (29.7%)	8 (38.1%)	77 (30.4%)
Age			
Mean (SD)	43.0 (11.4)	51.5 (12.2)	43.7 (11.7)
Median [Min, Max]	43.0 [21.0, 71.0]	54.0 [30.0, 74.0]	44.0 [21.0, 74.0]
TI‐RADS			
3	4 (1.7%)	0 (0%)	4 (1.6%)
4a	46 (19.8%)	4 (19.0%)	50 (19.8%)
4b	129 (55.7%)	0 (0%)	128 (50.6%)
4c	37 (15.9%)	1 (4.8%)	38 (15.0%)
5	8 (3.4%)	1 (4.8%)	9 (3.6%)
Missing	8 (3.4%)	15 (71.4%)	23 (9.1%)
Ultrasound abnormal node			
No	61 (26.3%)	3 (14.3%)	64 (25.3%)
Yes	171 (73.7%)	18 (85.7%)	189 (74.7%)
Ultrasound calcification			
No	121 (52.2%)	18 (85.7%)	139 (54.9%)
Yes	111 (47.8%)	3 (14.3%)	114 (45.1%)
Histology			
MTC	4 (1.7%)	0 (0%)	4 (1.6%)
PTC	228 (98.3%)	21 (100%)	249 (98.4%)
Lymph node			
Negative	109 (47.0%)	5 (23.8%)	114 (45.1%)
Positive	123 (53.0%)	16 (76.2%)	139 (54.9%)
Diameter			
Mean (SD)	0.958 (0.746)	1.38 (1.20)	0.982 (0.782)
Median [Min, Max]	0.800 [0.200, 5.50]	1.10 [0.100, 4.50]	0.800 [0.100, 5.50]
Missing	17 (7.3%)	8 (38.1%)	25 (9.9%)
Stage			
I	215 (92.7%)	4 (19.0%)	219 (86.6%)
II	14 (6.0%)	11 (52.4%)	25 (9.9%)
IVA	3 (1.3%)	2 (9.5%)	5 (2.0%)
IVB	0 (0%)	4 (19.0%)	4 (1.6%)
T stage			
T1a	179 (77.2%)	6 (28.6%)	185 (73.1%)
T1b	39 (16.8%)	4 (19.0%)	43 (17.0%)
T2	13 (5.6%)	3 (14.3%)	16 (6.3%)
T3a	1 (0.4%)	1 (4.8%)	2 (0.8%)
Tx	0 (0%)	7 (33.3%)	7 (2.8%)
N stage			
N0	110 (47.4%)	4 (19.0%)	114 (45.1%)
N1a	74 (31.9%)	0 (0%)	74 (29.2%)
N1b	48 (20.7%)	17 (81.0%)	65 (25.7%)
M stage			
M0	232 (100%)	5 (23.8%)	237 (93.7%)
M1	0 (0%)	16 (76.2%)	16 (6.3%)

### 3.2 Differences between patients with and without lymph node metastasis of primary cases

**FIGURE 2:**
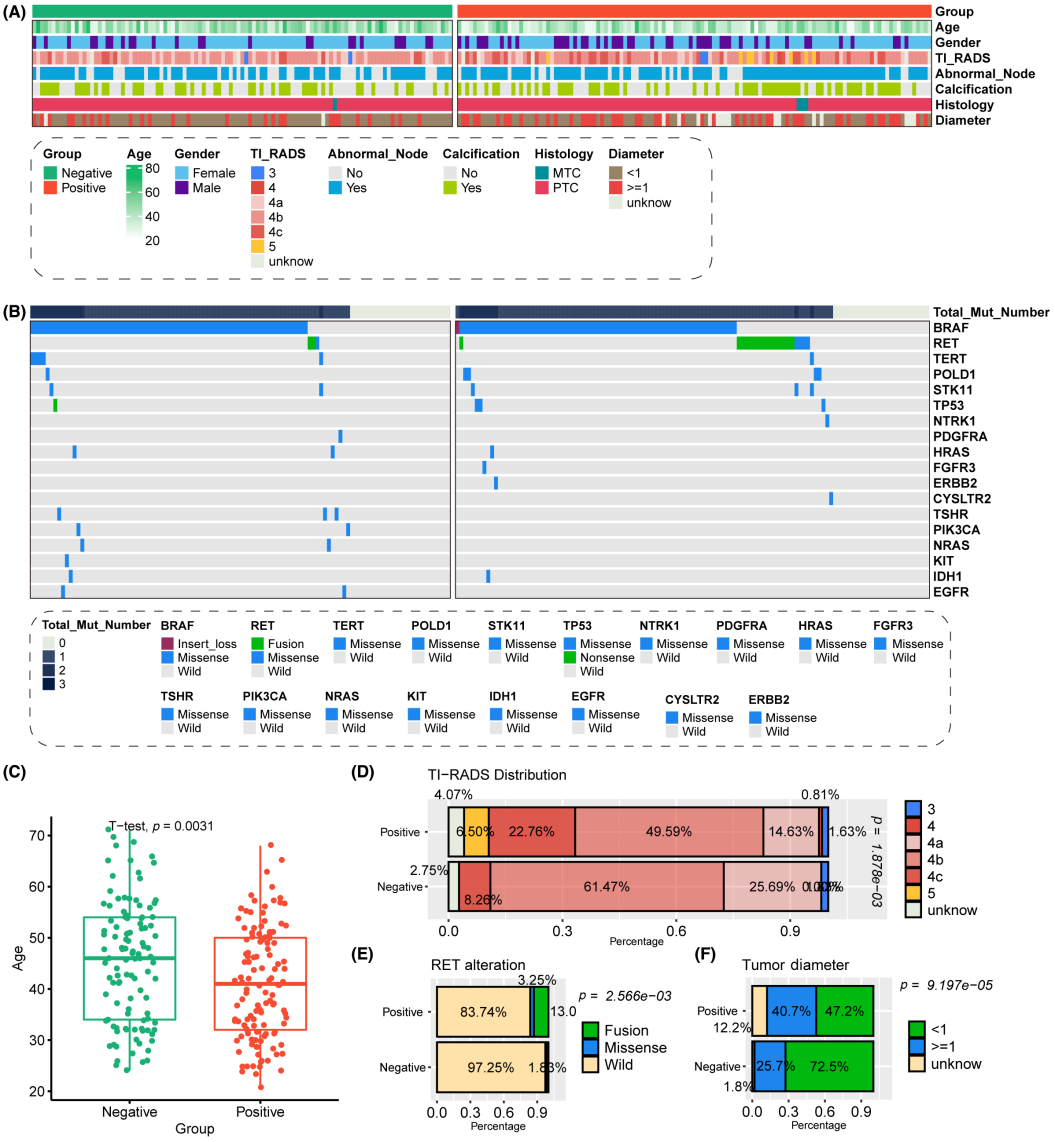
Analysis of differences in gene mutations and clinical indicators between patients with and without lymph node metastasis of primary cases. (A) Distribution of clinical and pathological information. (B) Differential distribution of gene mutations. (C) Diverse diagnosed patients' age. (D) Diverse ultrasound detected TI‐RADS level. (E) Differential distribution of RET genetic alteration. (F) Differential distribution of tumor longest diameter.

As we talked in the introduction part, lymph node metastasis is tightly with the prognosis of TC, therefore it is essential to identify the novel factors to help the identification of lymph node metastasis in clinical work. We analyzed the diverse distribution of clinical and pathological features among patients with and without lymph node metastasis from AHMU‐TC cohort (Table 2; Figure 2). The distribution of patients' sex is a little bit different, more than half enrolled male patients met lymph node metastasis (35.8% vs. 22.9%). Patients with positive lymph node were younger (40.9 ± 10.8 vs. 45.3 ± 11.8, *p* = 0.0031, Figure 2C) and contained advanced TI‐RADS levels (4c: 22.8% vs. 8.3%, 5: 6.5% vs. 0/0%, *p* = 1.878e‐03, Figure 2D). Moreover, we also found that patients with positive lymph node also had the larger tumor represented by the tumor diameter than those with negative lymph node ones (≥1 cm: 40.7% vs. 25.7%, *p* = 9.197e‐05, Figure 2F), as well as the presence of RET genetic alteration (16.3% vs. 2.7%, *p* = 2.566e‐03, Figure 2E).

**TABLE 2:** Different clinical features among patients with or without lymph node metastasis.

	Negative (*N* = 109)	Positive (*N* = 123)	*p* value
Gender			
Female	84 (77.1%)	79 (64.2%)	0.044
Male	25 (22.9%)	44 (35.8%)	
Age			
Mean (SD)	45.3 (11.8)	40.9 (10.8)	0.003
Median [Min, Max]	46.0 [24.0, 71.0]	41.0 [21.0, 68.0]	
TI_RADS			
3	2 (1.8%)	2 (1.6%)	<0.001
4a	28 (25.7%)	18 (14.6%)	
4b	67 (61.5%)	61 (49.6%)	
4c	9 (8.3%)	28 (22.8%)	
Unknown	3 (2.8%)	5 (4.1%)	
4	0 (0%)	1 (0.8%)	
5	0 (0%)	8 (6.5%)	
Abnormal Node			
No	35 (32.1%)	26 (21.1%)	0.073
Yes	74 (67.9%)	97 (78.9%)	
Calcification			
No	62 (56.9%)	59 (48.0%)	0.19
Yes	47 (43.1%)	64 (52.0%)	
Histology			
MTC	1 (0.9%)	3 (2.4%)	0.625
PTC	108 (99.1%)	120 (97.6%)	
Diameter			
<1	79 (72.5%)	58 (47.2%)	<0.001
≥1	28 (25.7%)	50 (40.7%)	
Unknown	2 (1.8%)	15 (12.2%)	
EGFR			
Missense	2 (1.8%)	0 (0%)	0.22
Wild	107 (98.2%)	123 (100%)	
IDH1			
Missense	1 (0.9%)	1 (0.8%)	1
Wild	108 (99.1%)	122 (99.2%)	
KIT			
Missense	1 (0.9%)	0 (0%)	0.47
Wild	108 (99.1%)	123 (100%)	
NRAS			
Missense	2 (1.8%)	0 (0%)	0.22
Wild	107 (98.2%)	123 (100%)	
PIK3CA			
Missense	2 (1.8%)	0 (0%)	0.22
Wild	107 (98.2%)	123 (100%)	
TSHR			
Missense	3 (2.8%)	0 (0%)	0.102
Wild	106 (97.2%)	123 (100%)	
CYSLTR2			
Wild	109 (100%)	122 (99.2%)	1
Missense	0 (0%)	1 (0.8%)	
ERBB2			
Wild	109 (100%)	122 (99.2%)	1
Missense	0 (0%)	1 (0.8%)	
FGFR3			
Wild	109 (100%)	122 (99.2%)	1
Missense	0 (0%)	1 (0.8%)	
HRAS			
Missense	2 (1.8%)	1 (0.8%)	0.602
Wild	107 (98.2%)	122 (99.2%)	
PDGFRA			
Missense	1 (0.9%)	0 (0%)	0.47
Wild	108 (99.1%)	123 (100%)	
NTRK1			
Wild	109 (100%)	122 (99.2%)	1
Missense	0 (0%)	1 (0.8%)	
TP53			
Nonsense	1 (0.9%)	0 (0%)	0.172
Wild	108 (99.1%)	120 (97.6%)	
Missense	0 (0%)	3 (2.4%)	
STK11			
Missense	2 (1.8%)	3 (2.4%)	1
Wild	107 (98.2%)	120 (97.6%)	
POLD1			
Missense	1 (0.9%)	4 (3.3%)	0.374
Wild	108 (99.1%)	119 (96.7%)	
TERT			
Missense	5 (4.6%)	1 (0.8%)	0.102
Wild	104 (95.4%)	122 (99.2%)	
RET			
Fusion	2 (1.8%)	16 (13.0%)	0.001
Missense	1 (0.9%)	4 (3.3%)	
Wild	106 (97.2%)	103 (83.7%)	
BRAF			
Missense	72 (66.1%)	72 (58.5%)	0.31
Wild	37 (33.9%)	50 (40.7%)	
Insert_loss	0 (0%)	1 (0.8%)	

### 3.3 Identifying of the independent prognostic factor and constructing the nomogram

**FIGURE 3:**
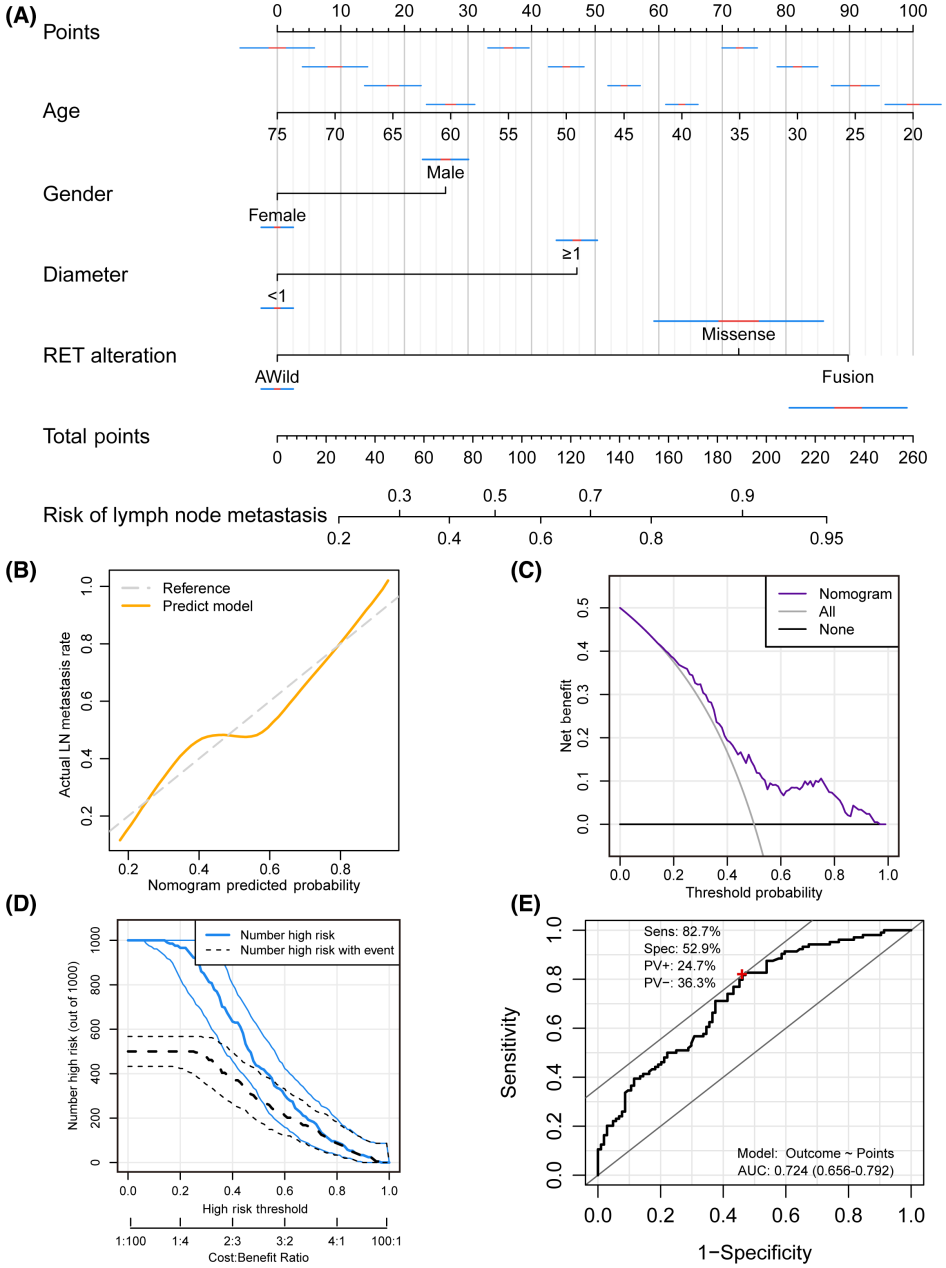
Construction of a nomogram for predicting lymph node metastasis and evaluating the clinical application of the nomogram. (A) Nomogram for the prediction of lymph node metastasis for thyroid cancer patients with the factors of age, gender, tumor diameter, and RET genetic alteration. (B) Calibration plot for the nomogram. (C) DCA showed that our nomogram has a greatest net benefit. (D) Clinical impact curve of the nomogram plots the number of lymph node metastasis patients classified as high risk, and the number of cases classified as high risk with the event at each risk threshold. (E) ROC curve showed the preferable prognostic value of the nomogram.

The factors that showed significantly difference between patients with/without lymph node metastasis, including age, gender, ultrasound defined TI‐RADS level, tumor diameter and RET genetic alteration, were enrolled for the multivariate logistic regression analysis (Table 3). We found that after adjusting the potential impact factors, patient age (*p* = 0.002), tumor diameter (*p* = 0.034), and RET fusion (*p* = 0.032) still as the prognostic factor to the presence of positive lymph node, while patient gender also showed a marginal prognostic value (*p* = 0.08). Therefore, we enrolled age, gender, tumor diameter, and RET alteration to construct the lymph node metastasis predict nomogram (Figure 3A). In the calibration plot, the nomogram predict result is tightly close to the refer line, which indicated that the prediction nomogram was an ideal predictive model (Figure 3B). DCA curve and the clinical impact curve was performed to demonstrate high clinical net benefit that almost over the entire threshold probability of the nomogram model (Figure 3C,D). To assess the predictive performance of the current model, we performed a ROC curve analysis, which exhibited an AUC value of 0.724 (0.656–0.792). The model's sensitivity and specificity were 82.7% and 52.9%, respectively, serving as a supplementary indicator to signal the probability of lymph node metastasis for patients (Figure 3E).

**TABLE 3:** Multivariate logistic regression analysis for the prediction of lymph node metastasis.

Characteristic	OR	95% CI	*β*	SE	Wald value	*p* value
Age	0.96	0.93, 0.98	−0.044	0.014	−3.062	0.002*
Gender						
Female	—	—	—	—	—	
Male	1.79	0.93, 3.49	0.584	0.335	1.742	0.081
TI_RADS						
3	—	—	—	—	—	
4a	0.69	0.07, 6.73	−0.369	1.099	−0.336	0.7
4b	1.14	0.12, 10.6	0.134	1.068	0.125	0.9
4c + 5	3.66	0.35, 38.5	1.299	1.139	1.14	0.3
Unknown	1.09	0.08, 15.9	0.089	1.315	0.068	>0.9
Diameter						
<1	—	—	—	—	—	
≥1	2.03	1.06, 3.95	0.710	0.335	2.121	0.034*
Unknown	10.9	2.73, 73.4	2.388	0.799	2.987	0.003*
RET						
Wild	—	—	—	—	—	
Fusion	5.83	1.44, 39.4	1.762	0.804	2.193	0.028*
Missense	4.43	0.55, 93.8	1.489	1.191	1.25	0.2

Abbreviations: CI, confidence interval; OR, odds ratio; SE, standard error for *β*.

* *p* < 0.05.

### 3.4 Validating the prognostic value of the nomogram in TCGA‐THCA cohort

**FIGURE 4:**
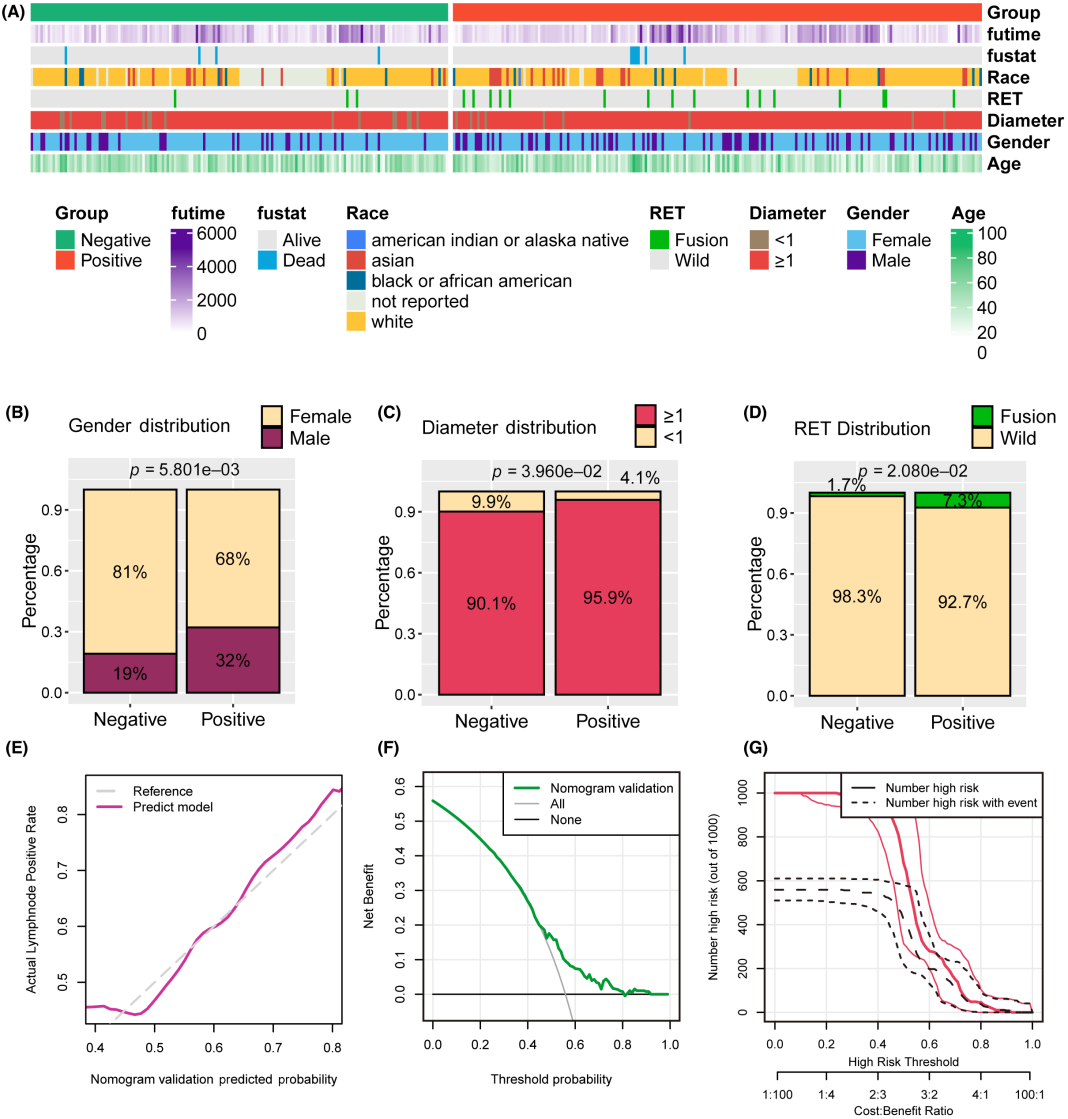
Evaluation of clinical information, RET fusions, and validation of the predictive power of nomograms between patients with lymph node metastases/non‐metastases in external TCGA‐THCA cohort. (A) Distribution of clinical and pathological information. (B) Differential distribution of patients' gender. (C) Differential distribution of tumor diameter. (D) Differential distribution of gene RET fusion. (E) Calibration plot for the nomogram. (F) DCA showed that our nomogram has a greatest net benefit. (G) Clinical impact curve of the nomogram plots the number of lymph node metastasis patients classified as high risk, and the number of cases classified as high risk with the event at each risk threshold.

We collected the clinicopathological features and gene mutation matrix of 390 TC patients from TCGA‐THCA cohort to validate the prognostic value of the nomogram, these features displayed in Figure 4A. We observed that more males met the positive lymph node after operation (32.0% vs. 19.0%, *p* = 0.0058, Figure 4B), which is consistent with our own cohort. Moreover, patients with positive lymph node also had the larger tumor represented by the tumor diameter than those with negative lymph node ones (≥1 cm: 95.9% vs. 90.1%, *p* = 0.0396, Figure 4C), as well as the presence of RET genetic alteration (7.3% vs. 1.7%, *p* = 0.0208, Figure 4D). In the calibration plot, the nomogram predict result is tightly close to the refer line, which indicated that the prediction nomogram was an ideal predictive model (Figure 4E). DCA curve and the clinical impact curves was performed to demonstrate high clinical net benefit that almost over the entire threshold probability of the nomogram model (Figure 4F,G).

### 3.5 Discover the potential mechanism of how RET fusion impact lymph node metastasis

**FIGURE 5:**
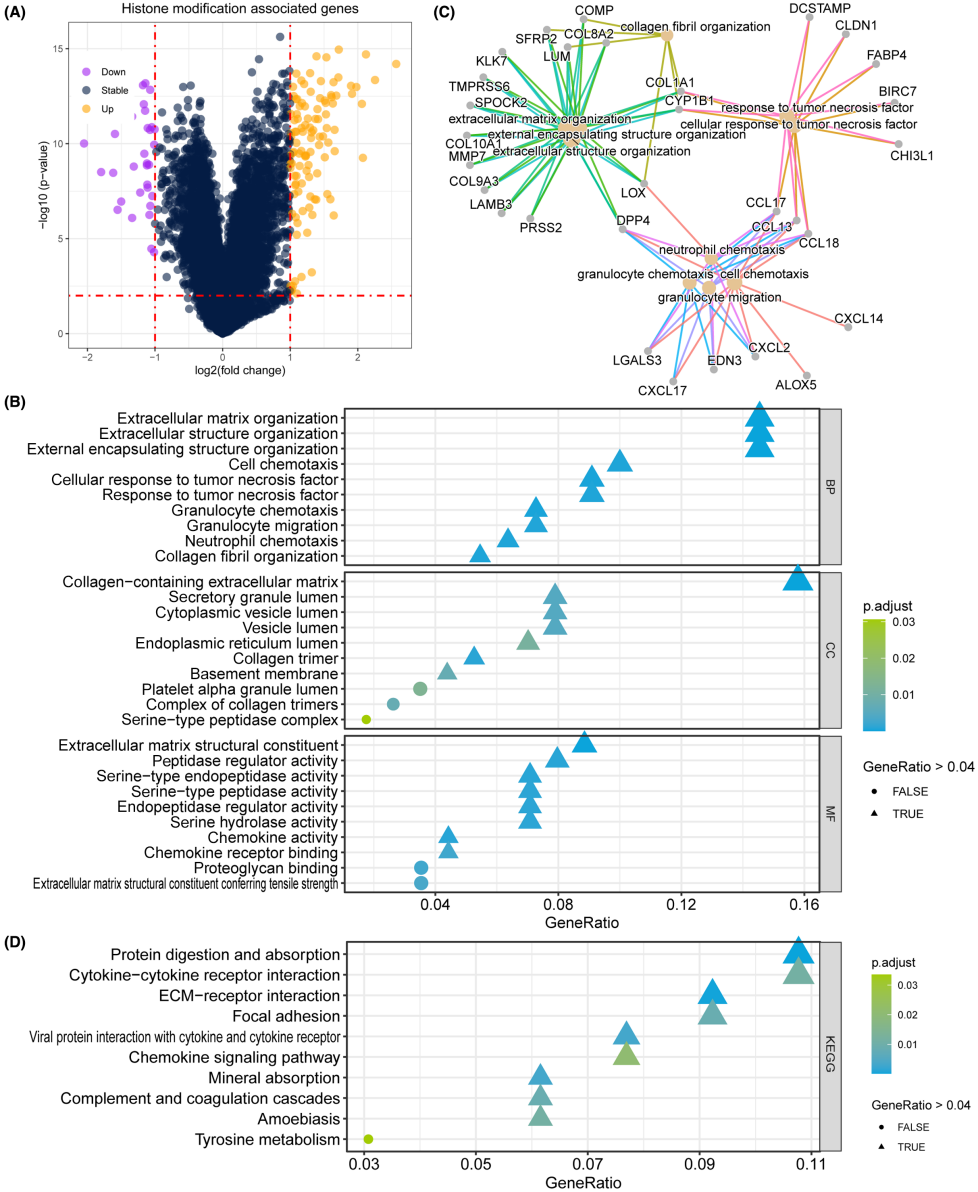
Differentially expressed genes and activated pathways between RET fusion/unfusion patients to explore the potential mechanism between RET fusion and lymph node metastasis. (A) Volcano plot showing the differentially expressed genes. (B) RET fusion alteration leading the activation of biological process. (C) Network plot showing the connection of top 10 impact pathways.

To reveal the potential correlation between RET fusion and TC patient lymph node metastasis, we collected the gene expression profile of the TCGA‐THCA cohort. We compared the different expression of genes, and found 102 unregulated gene in RET fusion samples, and 31 gene upregulated in samples with RET fusion (Figure 5A). And we subsequently annotated these 133 DEGs, and revealed that RET fusion mostly regulated the activation of extracellular matrix and structure organization, chemokine receptor binding (Figure 5B), and the network indicate that the key genes are LOX, DPP4, CCL17, CCL13, CCL18, CYP1B1, COL8A2 (Figure 5C). The KEGG pathway enrichment also revealed that RET fusion impact the process of protein digestion and absorption, cytokine‐cytokine receptor interaction, ECM‐receptor interaction, focal adhesion (Figure 5D).

### 3.6 A scheme of nomogram application

To enhance the practical applicability of the nomogram, we provide a specific example to guide its use in clinical predictions. For instance, in the case of a 53‐year‐old male thyroid cancer patient, we discovered that the tumor diameter exceeds 1 cm and presents a RET mutation. Consequently, according to the nomogram, we can identify the points corresponding to each parameter, which are 26, 40, 47, and 72. By summing these points, we obtain a total of 185 points, corresponding to an approximate probability of 0.88 for the occurrence of lymph node metastasis.

## 4 DISCUSSION

The increase in the incidence of thyroid cancer presents a “wave‐like” trend, its detection rate is increasing all over the world, and its diagnosis is getting younger and younger. The current mainstream view is that due to the high sensitivity and strong accuracy of high‐resolution ultrasound diagnostic technology, smaller and earlier thyroid nodules are continuously detected, and thyroid cancer accounts for about 5%–15% of them.^21^ At the same time, obesity, estrogen, radiation exposure, iodine intake, and other factors, have led to the high incidence of thyroid cancer in recent years.^22^, ^23^, ^24^

The occurrence and development of TC is a process involved multiple genetic alterations, including gene mutations, rearrangements, fusions, and methylation of oncogenes and tumor suppressor genes. PTC is the most common type of thyroid cancer, although a better outcome can be expected for PTC patients, 9.1%–38% of them will finally step into the status of lateral lymph node metastasis, which seriously affects the prognosis.^25^ Therefore, it is necessary to identify the risk factors of lymph node metastasis for TC patients, to distinguish patients with high risk of poor prognosis and give the aggressive treatment.

The product encoded by the BRAF gene is a protein kinase that regulates cell differentiation, reproduction, and apoptosis, and affects the carcinogenesis of the thyroid through the RAS‐RAF‐MEK‐ERK‐MAPK signaling pathway.^26^ According to the report of The Catalog of Somatic Mutations in Cancer database, the researchers collected nearly 80,000 cases of BRAF gene mutation, and found that BRAF^V600E^ mutation ranks first among all types, accounting for more than 95%.^27^ In the current study, 65.21% patients were detected with BRAF mutations, 62.07% for primary cases, and 90.48% for secondary cases. BRAF mutation seems correlated with the recurrent of TC. Another study based on east China also reported that BRAF mutation is correlated with larger tumor size, higher probability of PTC recurrence and LNM.^28^ However, we failed to observe the correlation of BRAF mutation with LNM. A meta‐analysis reported that LNM is not associated with BRAF mutation of PTC patients.^29^

The RET proto‐oncogene encodes a transmembrane glycoprotein receptor with tyrosine kinase activity, of which participates in the processes of proliferation, differentiation and motility.^30^ The incidence of RET/PTC rearrangement is about 15%–20% in sporadic PTC, and the positive rate of RET gene mutation is nearly 50% in patients with medullary thyroid cancer. In people with clear radiation exposure, the positive rate of RET gene mutation can be as high as 65%. In the current study, we observed 23 cases contained the genetic alteration of RET, including 18 fusion and 5 missense mutation, all these genetic alterations presented in the primary cases, but no one in secondary cases. Furthermore, we also observed that RET fusion is the independent predictor for LNM, but not the missense mutation. Another clinical study from east China reported 10.4% RET fusions in 193 PTC patients,^31^ 7.08% RET fusion reported in a study from 14 tertiary hospitals of China.^32^ Ullmann et al.^33^ also reported that RET‐driven tumors are more likely to have extrathyroidal extension, multifocal disease and distant metastases in American.

Drawing on the data from the 232 patients, we observed that age, gender, and tumor diameter exhibited predictive value for LNM, with early age, male gender, and a diameter greater than 1 cm appearing to be risk factors. Evidence from other studies supports our findings. A study from central China discovered that patients aged ≤18 years (OR = 4.41, *p* < 0.001) and 19–45 years (OR = 1.97, *p* = 0.002) had a higher risk of lateral LNM than patients aged >60 years.^34^ A systematic review and meta‐analysis encompassing 27,741 patients from 41 studies indicated that age < 45 years and male gender were risk factors for central lymph node metastasis.^35^ A 2010 study based on a Korean population found that tumor diameter >2 cm was significantly associated with lymph node metastasis,^36^ while a 2012 Japanese study supported this finding, stating that tumor size (>2 cm) was the strongest predictor of microscopic central and lateral node metastasis in multivariate logistic analysis. Moreover, tumor size most markedly impacted lymph node recurrence, but not distant recurrence.^37^ Considering the widespread use of Doppler ultrasound in the early diagnosis of TC, an increasing number of TC cases are being detected at an early stage. Consequently, current research should focus more on the impact of small tumor sizes on clinical prognosis, such as the <1 cm threshold established in this study.

Based on the identified prognostic factors, age, gender, tumor diameter, and RET alteration, we constructed the LNM prediction nomogram, validated by the calibration, DCA, and clinical impact curve, all the results indicated that the nomogram presenting a high accuracy. What's more, we also validated the predict function of the nomogram based on the data from TCGA‐THCA cohort. For the potential underlying mechanism of how RET fusion impact LNM, we analyzed the DEGs and annotated, which pointed out that RET fusion might impact LNM through the regulation of cytokine‐cytokine receptor interaction, ECM‐receptor interaction and focal adhesion. We should focus on the advantages of the current study. First, we collected 253 TC patients and recorded the clinical information, pathological information and results of NGS. Second, we developed a LNM prediction nomogram based on the age, gender, tumor diameter, and RET alteration. Third, we successfully validated the prediction accuracy of the nomogram in external cohort. There are still several limitations of the current study. First of all, this study is a single center study, patients from multiple medical centers are necessary to collected to further validate the nomogram. In addition, the mechanism of how RET fusion promote LNM is limited, further experiment is needed in the future study, the current study is just a small step.

## 5 CONCLUSION

We provide a novel nomogram to predict the LNM for TC patients, concerns the features of patient's age, gender, tumor diameter, and RET alteration. Further studies from multiple medical centers are essential to validate the predict stability and accuracy of the nomogram.

## AUTHOR CONTRIBUTIONS

**Meng Wei:** Conceptualization (equal); investigation (equal); visualization (equal); writing – original draft (equal). **Rui Wang:** Data curation (equal); formal analysis (equal). **Wanxue Zhang:** Resources (equal); software (equal); visualization (equal). **Jing Zhang:** Investigation (equal); validation (equal); visualization (equal). **Qiang Fang:** Resources (equal); supervision (equal); validation (equal); writing – original draft (equal). **zheng fang:** Investigation (equal); methodology (equal). **Bin Liu:** Investigation (equal); supervision (equal); writing – review and editing (equal). **Yongxiang Li:** Conceptualization (equal); data curation (equal); writing – review and editing (equal).

## ETHICS APPROVAL AND CONSENT TO PARTICIPATE

The research contents and research programs were reviewed and approved by the Ethics Committee of the First Affiliated Hospital of Anhui Medical University (Quick‐PJ‐2022‐13‐44).

## Supporting information


Figure S1.

## Data Availability

The data that support the findings of this study are available upon reasonable request from the corresponding author.
