# Supplementary figures and images for: Landscape of gene mutation in Chinese thyroid cancer patients: Construction and validation of lymph node metastasis prediction model based on clinical features and gene mutation marker
Source: Cancer Med. 2023 Apr 20;12(11):12929–42. doi: 10.1002/cam4.5945 (PMC10278465; doi:10.1002/cam4.5945)

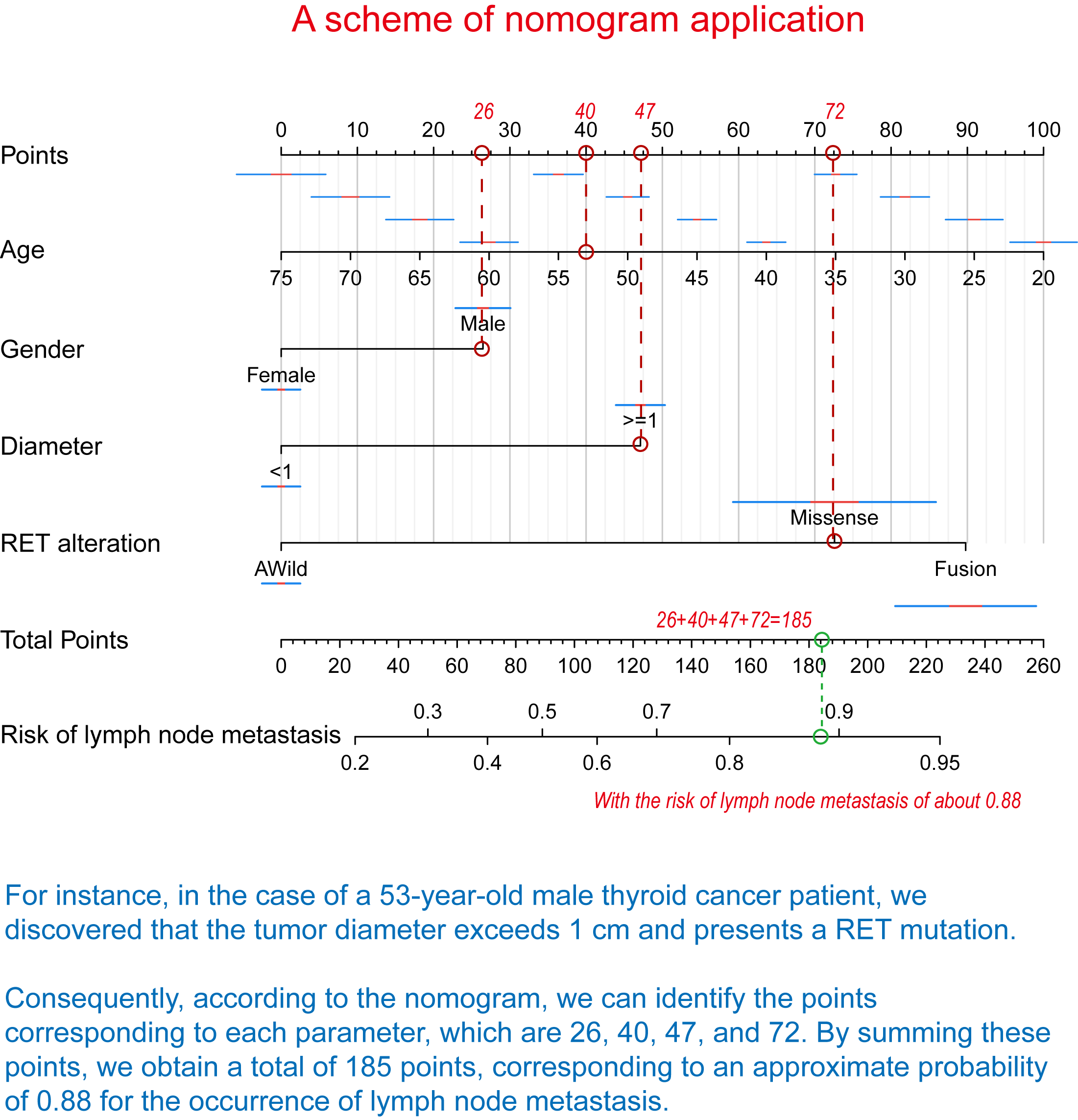

Supplement: Supplementary file 1 — Figure S1. [file CAM4-12-12929-s001.tif]
